# The Advent of the Internet of Things in Airfield Lightning Systems: Paving the Way from a Legacy Environment to an Open World

**DOI:** 10.3390/s19214724

**Published:** 2019-10-31

**Authors:** Enrico Buzzoni, Fabio Forlani, Carlo Giannelli, Matteo Mazzotti, Stefano Parisotto, Alessandro Pomponio, Cesare Stefanelli

**Affiliations:** 1 R&D Department, OCEM Airfield Technology, BO 40056 Crespellano, Italy; enrico.buzzoni@ocem.com (E.B.); matteo.mazzotti@ocem.com (M.M.); 2 Department of Mathematics and Computer Science, University of Ferrara, FE 44124 Ferrara, Italy; fabio01.forlani@student.unife.it (F.F.); stefan.parisotto@student.unife.it (S.P.); 3 Department of Computer Science and Engineering, University of Bologna, BO 40100 Bologna, Italy; alessandro.pomponio2@studio.unibo.it; 4 Department of Engineering, University of Ferrara, FE 44124 Ferrara, Italy; cesare.stefanelli@unife.it

**Keywords:** Internet of Things, middleware, airfield lightning

## Abstract

This paper discusses the design and prototype implementation of a software solution facilitating the interaction of third-party developers with a legacy monitoring and control system in the airfield environment. By following the Internet of Things (IoT) approach and adopting open standards and paradigms such as REpresentational State Transfer (REST) and Advanced Message Queuing Protocol (AMQP) for message dispatching, the work aims at paving the way towards a more open world in the airfield industrial sector. The paper also presents performance results achieved by extending legacy components to support IoT standards. Quantitative results not only demonstrate the feasibility of the proposed solution, but also its suitability in terms of prompt message dispatching and increased fault tolerance.

## 1. Introduction

Traditional monitoring and control systems have been characterized by closed and proprietary solutions designed and developed from scratch by enterprises. In fact, the lack of established and widely recognized standards supporting the interaction with devices have led to a push for a plethora of heterogeneous and highly tailored solutions addressing the issues of specific environments [1]. 

This approach has provided some notable advantages. Enterprises have full control of the developed system and the adopted protocols, since they are based on code and specifications designed and written with limited (or no) adoption of external software libraries and standards. In this manner, there are no issues related to licenses or modification of third-party code. In addition, enterprises have the perception that they have a more secure solution, since only internal developers know its source code and potential attackers cannot exploit the known bugs of libraries and third-party services.

However, these closed and proprietary solutions have demonstrated that they cannot keep up with technology evolution in terms of, e.g., software complexity and increased device heterogeneity, and tend to present three notable shortcomings. First of all, the development of new systems and solutions is highly complex, making it difficult to directly manage details ranging from the interaction with devices to the formatting and transmission of data, its storing and presentation. Secondly, monitoring and control solutions are usually part of wider solutions that also comprise other aspects of the whole target environment. In such a case, the cost of integrating different proprietary solutions would be very high. Finally, the integration with third-party systems has to also consider security concerns related to the enforcing of authentication and authorization policies to ensure that only legitimate users and code can access data and run, respectively. Developing security mechanisms from scratch can be a very challenging task, eventually posing serious threatens in the case of bugs or misconfigurations (as demonstrated by the Stuxnet industrial malware [2]). 

The recent advances in the Information Technology (IT) field have provided novel development opportunities based on de facto standards, which emerged thanks to their easy adoption in heterogeneous environments. Enterprises are more and more commonly required to adopt such an open approach to support easy integration of their solutions with third-party systems as well as to develop new custom features in addition to already supported ones, all in a secure way. In particular, the widespread adoption of the Internet of Things (IoT) paradigm demonstrates the feasibility of easily integrating any physical or virtual component [3], allowing us to add new functionalities on-top-of already developed ones. In addition, the adoption of well-known and widely used/tested software libraries increases the level of trustiness of the whole system. 

The design and development effort presented in the paper (a joint effort of OCEM Airfield Technology and the University of Ferrara) represents a notable example of how it is possible to pave the way from proprietary to open IoT-based solutions to monitor and control lights and power supplies for civil and military airports. In other words, the paper shows how the adoption of IoT enabling technologies can support data collection and increase interoperability of software components in a mission-critical IoT application, also allowing for more easy interaction with sensors and actuators deployed in the airfield. Traditionally, lighting monitoring systems in airfield environments are based on reliable, high-quality Airport Ground Lighting (AGL) and Airport Lights Control and Monitoring System (ALCMS) solutions. Eventually, additional software components allow third-party airport software applications to interact with the airport lighting system, e.g., to gather airport weather data and compute the Runway Visual Range (RVR) value (FAA explanation of RVR available at https://www.faa.gov/about/office_org/headquarters_offices/ato/service_units/techops/navservices/lsg/rvr/). Starting from traditional AGL and ALCMS solutions based on proprietary protocols, the paper reports on the work recently done to adopt the IoT paradigm and related standards, such as well-known REpresentational State Transfer (REST) [4] and Advanced Message Queuing Protocol (AMQP) [5] protocols. The proposed solution permits us to exploit the already developed and widely tested airfield lightning system as is, thus avoiding the burden of modifying the legacy software itself, while providing previous and novel features in a standard way. The IoT paradigm has greatly improved the easy management of the solution from a code maintenance point of view, also allowing us to more easily developing novel features, while the increased openness of the monitoring and control system makes the integration with third-party systems easier.

## 2. Related Work

Years before the advent of IoT, the computer-based remote monitoring of airfield lightning systems was already a well-known and widely adopted solution that was useful for improving airport efficiency and safety [6,7]. For instance, reference [6] represents a seminal work proposing to adopt an integrated communication network to monitor the airfield lightning system, stating that such a solution can improve air control efficiency, aircraft safety, and efficiency of ground operations. However, traditionally proposed solutions for airfield lighting management rely on proprietary software components directly exploiting low-level communication features provided by operating systems, even in case of relatively recent cases. For instance, reference [8] exploits client/server architecture dispatching data via TCP/IP sockets, by adopting multithreading and timeout techniques to improve efficiency. Let us stress that such a solution only focuses on reliability and responsiveness as objectives, while neglecting issues related to easy maintenance, extendibility, and integrability. In reference [9] an innovative solution based on wireless sensor networks (WSNs) was proposed to monitor airfield lightning systems, by verifying its lifetime in relation to per-node energy consumption. However, the solution has only been verified in a simulated environment. Other recent proposals in the airfield scenario focused on upgrading the lighting system, e.g., from incandescent- to led-based lights to improve lifetime and reduce energy consumption while ensuring a uniform light output [10]. 

The airport environment has fostered the adoption of more articulated and advanced networking solutions, but mainly for non-mission-critical use cases. For instance, reference [11] and reference [12] exploit RFID tags to track baggage within airports to improve both baggage management efficiency and security. Other innovative services proposed for airport environments provided a solution to predict queue time based on the IEEE 802.11 positioning system [13], the real-time monitoring of indoor air quality [14], and a WSN-based safety monitoring system [15]. In contrast, outside the airport environment, several lighting management systems have been proposed, but with little or no mission critical requirements. For instance, reference [16] proposes an IoT lighting system that can be exploited as a central part of connected buildings. 

The IoT approach has also been recently proposed in other mission-critical environments, e.g., to support the real-time condition monitoring and the fast detection of faults in the water industry [17], to adopt unmanned aerial vehicles to monitor concrete plants [18], and to maximize the quality of service in operating rooms to deliver vital medical services in hospitals [19]. Considering the transportation field, [20] presents an IoT-based smart maintenance solution for railway systems comprising device platforms, gateways, and IoT servers. Reference [20] focuses on the performance and cost comparison of different wireless technologies, justified by the fact that the geographical scope of a railway is really wide and monitored railway devices are deployed in a sparse manner. 

Overall, solutions aiming at remotely monitoring airfield lighting systems usually focus on fault tolerance and responsiveness only, while neglecting extendibility and interoperability. More articulated solutions in airport environments have been recently proposed, but only for non-mission-critical use cases. Other innovative IoT solutions for lightning systems are available, but for smart buildings and smart cities, which are characterized by more advanced technology environments and with limited requirements in terms of fault tolerance and responsiveness compared with airfield environments. Finally, other solutions propose novel IoT-based monitoring and control solutions in different mission-critical environments, such as for hospitals and railway systems.

## 3. Materials and Methods

### 3.1. Technology for Airfield Monitoring and Control

**Figure 1:**
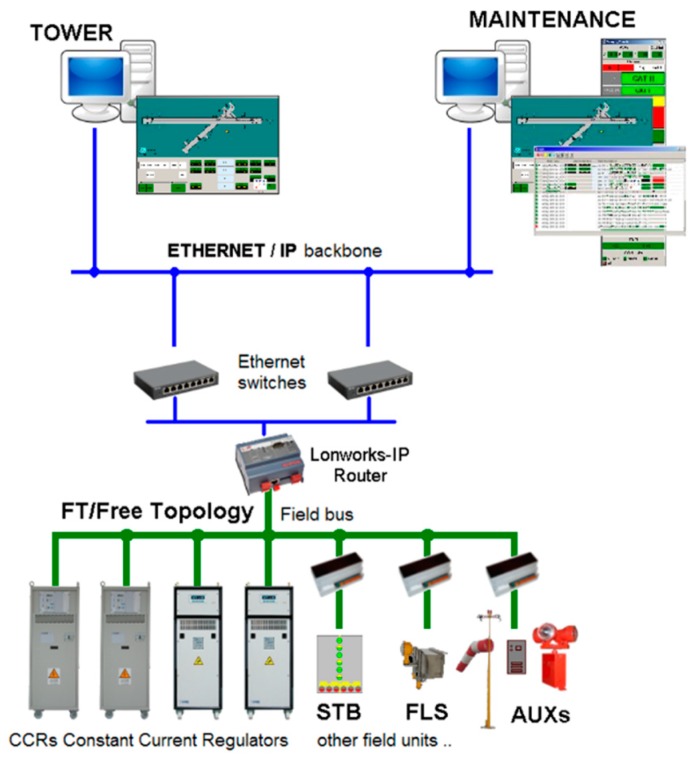
An example of Airport Lights Control and Monitoring System (ALCMS) network architecture.

OCEM follows and adopts airport international specifications such as the ALCMS Federal Aviation Administration (FAA) L-890 [21]. The current ALCMS implementation is based on a multi-layer network architecture exploiting different protocols to manage and supervise specific hardware units (Figure 1). 

At the bottom layer reside components directly supervising lights and other hardware devices usually communicating with the LonWorks industrial protocol (standard ANSI/CEA 709.1 [22]):

- Constant current regulator (CCR) units, power suppliers of the lighting system serial circuits devoted to, e.g., runways, PAPI (precise approach path indicators), and taxiways. Functional redundancy is guaranteed by inter-leaving lights connected to electrical circuits energized by independent regulators;
- sensor (SNS) nodes, supervising transit sensors of specific areas or taxiway junctions to detect the passage of airplanes and to advice in case of unauthorized access to runway (incursion alarm);
- Stop bar (STB) nodes, coordinating CCR and SNS nodes to manage segments of the lighting system with the role of semaphores, barriers, and path-to-runway lights;
- auxiliary (AUX) nodes, managing multipurpose contacts used to monitor and control in-field specific functions adopting an on/off logic, e.g.,:
  ○ monitoring of electric units and uninterruptible power supply (UPS) devices;
  ○ turning on/off devices, e.g., aerodrome beacons and wind cone lights;
  ○ monitoring general and customizable alarms gathered from specific hardware;
- flash (FLS) units, managing sets of periodically pulsing lights that indicate the runway approach path.

At the middle layer reside LonWorks-IP routers and IP switches that are in charge of providing IP access to the devices residing in the LonWorks network, thus connecting the ethernet backbone to LonWorks field buses.

At the top layer reside workstations hosting ALCMS/AGL tower and maintenance applications, which support human operators residing in the airport control tower and in the electric facilities respectively. Primary software components are:

- AGLcons, providing the main graphical user interface (GUI) to monitor and control the lighting system. It is worth noting that, to avoid potential command conflicts, a command mastership has to be a handshake between tower and maintenance substations;
- logger, tracing any system event provided by AGLcons and usually available only on the maintenance PC. Events may represent state information related to the bottom layer nodes and operative commands.

The AGL/ALCMS applications have been originally designed and developed as a close and proprietary solution accessible from human operators via the GUI. However, to allow the integration of third-party systems, we previously developed the application field server (AFS) solution to receive monitoring information and send actuation commands. AFS is based on a proprietary one-to-many socket-based communication component acting as intermediary among AGLcons and external clients. This component allows us to specify a set of clients and to serve multiple clients at the same time, e.g., to notify the same event to multiple clients or to dispatch a command to several units. For instance, the AFS interacts with the CCR unit to turn on/off light circuit power as well as to deliver primary alarms such as “open circuit” and “overcurrent”.

AFS notifies any event related to field units to every registered client. It is worth noting that AFS does not adopt any topic-based communication channel. In other words, CCR and AUX events are sent to every registered client, without any capability of differentiating which information a client is actually interested in, e.g., either CCR or AUX or a given category of CCR/AUX devices.

### 3.2. The Need for Open Airfield Solutions

Proprietary ALCMS-like architectures often presented the typical limits of solutions designed and developed when system integration was seen with skepticism, while the closedness of enterprise information systems was considered to be a standard approach. For instance, the AFS, introduced to permit third-party integration, was designed to operate only in closed networks, with the main objective being data raw dispatching, i.e., providing ALCMS packets as is with limited support to other non-functional features such as load balancing and fault tolerance. From a software engineering point of view, the ALCMS software components were developed from scratch, in part due to the limited performance capabilities of embedded systems, in part because suitable libraries were not available at all.

Changing customers’ requests and the evolution of software technologies pushed AGL manufacturers to revise their ALCMS solutions, moving towards open systems to ease the integration with third-party customer information systems and provide new functional (and non-functional) features, while improving the process of software production and maintenance. There is in fact an ever-increasing interest in developing custom features required by premium customers. To achieve this purpose, the exploitation of open and standard protocols lowers the effort required from third-party developers to integrate the proposed solution, thus making it more attractive from a market point of view.

Based on these considerations, we have identified as a primary requirement that already available features should be made accessible through models and protocols widely adopted and accepted by software developers. In this manner, the proposed solution keeps exploiting the already developed and widely tested legacy integration system while exceeding its limits and lowering its integration complexity. 

In particular, ALCMS information should be accessible in both request/response and publish/subscribe interaction models. The former is in charge of supporting clients of querying AFS when retrieving current and historical information. To this purpose, (part of) the information provided by AFS should be persisted in order to access them later both selectively and aggregately. For instance, persisted information could be retrieved by analytical tools to recognize recurring patterns of failures or misbehaviors, thus providing useful hints to identify faulty devices as well as further improve the whole monitoring and control system. For this purpose, the request/response interaction model should help well-documented application programming interfaces (APIs) to retrieve data segmented along several directions, e.g., by device, node type, airport area, and time period.

The latter is in charge of providing clients with up-to-date information as soon as they become available, dispatching important data in an event-driven fashion. In this manner, clients would be able to promptly react to state changes of the lighting system, e.g., rising an alarm in case an airplane enters a runway without authorization. Moreover, the publish/subscribe interaction model should be enhanced by allowing us to distribute information via topics, e.g., segmenting packets based on scope and interest. A client could be interested in information related to only runways while neglecting other areas of the airport lighting system. For instance, to compute the RVR value, only “runway center line” and “runway edge” CCRs are of interest.

Finally, it is worth noting that while opening the AFS solution to third-party developers increases its attractiveness, it also poses new challenges and issues that have to be thoroughly addressed in terms of availability, scalability, and security. To this purpose, first of all AFS should be accessible via replicated software architecture, thereby providing request/response and publish/subscribe access via independent and equipollent entry-points (thus achieving both load-balancing and fault tolerance). Secondly, to ensure graceful scalability of the whole architecture, it should be possible to flexibly introduce new communication components at service provisioning time, e.g., by allowing the deployment of new publish/subscribe broker replicas. Finally, security should be managed not only in terms of privacy (by adopting TLS encrypted communication whenever suitable) and authentication (by verifying the identity of each connecting client), but also enforcing authorization procedures (ensuring that authenticated clients can access only the part of data they are allowed to) and assessing the security of third-party software libraries. 

### 3.3. Web Technologies towards an Open World

When scouting for the software technologies best suited to our needs, we had the possibility to leverage on the huge work done in the IoT scenario and its related Web technologies, in particular REST and AMQP, two of the most interesting protocols for request/response and publish/subscribe interaction paradigms, respectively. Let us stress that the adoption IoT technologies, such as REST and AMQP protocols as well as OData Web API and JSON formatting standards (see Section 3.4), makes the proposed solution attractive for third-party developers, already accustomed to their use, while the availability of widely maintained libraries makes their adoption even easier. Moreover, IoT technologies allow us to easily integrate additional and heterogeneous devices, even after system deployment.

The REST paradigm allows us to interact with remote systems using any network environment that allows Web traffic, by mapping GET/POST/PUT/DELETE HTTP actions to stateless read/create/modify/delete operations. AMQP is an open Internet publish/subscribe messaging protocol that is recognized as an international standard [5] and supports both topics and queues, allowing us to dispatch the same packet to multiple subscribers simultaneously as well to only one of the available subscribers, respectively. Readers interested in additional information on REST, AMQP, and other IoT-related protocols are encouraged to read [23].

To increase availability, REST access should be provided by replicated Web servers while AMQP access via clustered brokers. In the former case, the request/response and stateless nature of the REST protocol allows the management of each Web server replica in a completely independent manner. In the latter case, AMQP broker replicas must be clustered to ensure that an event published by AFS via a broker is correctly delivered to any interested subscriber, despite the subscriber being connected to the same broker replica or another one. For this purpose, we adopted the ActiveMQ open source solution [24], a full-fledged multiprotocol broker that also supports several features for clustering and security.

Finally, let us note that while developing a complex monitoring and control system from scratch can be very challenging and prone to errors (thus threatening the security of the whole system), adopting open source libraries and solutions can also represent a security issue. In fact, even if developed and verified by several contributors, open source projects cannot be granted as bug-free. To this purpose, in addition to usual security best practices (e.g., nodes must run only strictly required services and with minimum required privileges on hardware and software resources), there is the need of periodically checking the availability of new versions of adopted libraries and software, with the primary goal of updating them whenever security bug fixes are provided. To this purpose, the adoption of the OWASP Dependency Check project [25] can be very beneficial, allowing the verification of the security of software (and, most relevant, also its dependencies) in relation to known vulnerabilities.

### 3.4. The OCEM Open System Solution

Based on the guideline and requirements presented above, we have designed a new and open ALCMS solution, whose core is a working prototype providing access to AFS features in an open manner based on REST and AMQP protocols.

**Figure 2:**
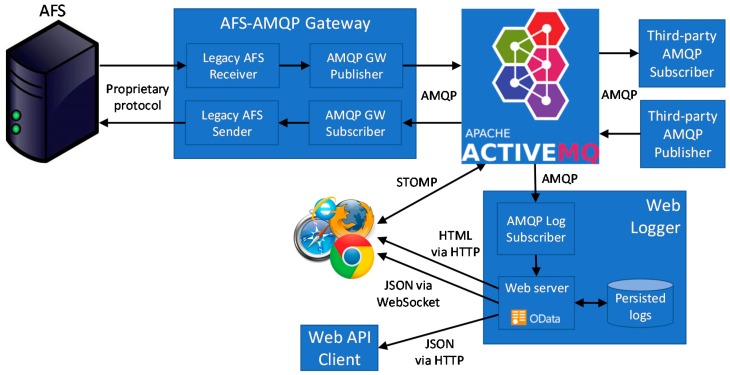
Overall architecture of the proposed solution.

Figure 2 presents the overall architecture of the proposed solution, based on two main components: the AFS-AMQP Gateway and the Web Logger. The AFS-AMQP Gateway represents the core component, in charge of dispatching messages between the legacy AFS application and ActiveMQ acting as the AMQP publish/subscribe broker. In particular, the AFS-AMQP Gateway acts as application double gateway, hiding the legacy AFS solution and providing previously available and new features in a transparent manner. On the one hand, it supports monitoring features by receiving from AFS any ALCMS event and then publishing it to ActiveMQ in AMQP format. On the other hand, it supports actuation by subscribing to ActiveMQ to receive commands sent by third-party clients and then sending them to AFS. 

It is worth noting that when receiving events from AFS, it publishes them via different topics. The current version of the AFS-AMQP Gateway exploits MNT.CCR/MNT.STB unit-related topics to dispatch monitored events related to constant current regulator and stop bar nodes, respectively (additional node-specific topics can be easily added whenever required). In addition, the same message is published to another topic related to the area of the airport the monitored device is related to, e.g., MNT.RWY for runways and MNT.TWY for taxiways, while commands are provided via the CMD topic.

The Web logger is in charge of gathering, persisting, and providing a request/response to any monitored event. To achieve this purpose, it subscribes to the ActiveMQ broker on the MNT.# topic to receive any event generated by AFS and published by the AMQP GW Publisher. For each event it receives, it interacts with a Web server to create a new entry in a SQL database (we currently exploit MariaDB, but it can be easily replaced with any SQL database). The Web server provides persisted information to any client requiring it. In particular, the Web server supports both REST-based Web API and an HTML-based GUI. In the former case, the process requiring the information is a REST client and the response is provided in JSON format by following the OData Standard for Open Data Exchange [26]. In the latter case, the process requiring the information is any standard Web browser and the response is provided in HTML format, thus allowing us to graphically display events in a human readable manner. 

Furthermore, the Web browser can directly connect to the ActiveMQ broker to subscribe to topics (as well as to publish messages) by exploiting the Simple Text Oriented Messaging Protocol (STOMP) protocol [27]. In this manner, it is possible to promptly push important messages, e.g., alarms, to Web app users. Otherwise, the Web client should periodically interact with the Web server to pull (eventually new) alarms, imposing network overhead as well as increased delay.

However, network administration policies could allow only HTTP traffic and prevent Web browsers from the possibility of creating connections to arbitrary TCP ports, such as the 61,613 default STOMP one. To overcome this issue, we also provided an alternative solution. In particular, the Web server supports the capability of upgrading HTTP connections to WebSockets, usually allowed by network security policies, to push important information to Web browsers in an event-driven manner. In this case, the Web Logger acts as a protocol gateway by translating AMQP messages in WebSocket ones.

## 4. Results and Discussion

We have developed and tested a working prototype of the proposed architecture with two objectives. On the one hand, our aim was to demonstrate the possibility of extending the legacy solution by exposing its features with standard IoT protocols. On the other hand, our aim was also to assess the soundness of adopted IoT technologies in terms of responsiveness, reliability, and scalability.

**Figure 3:**
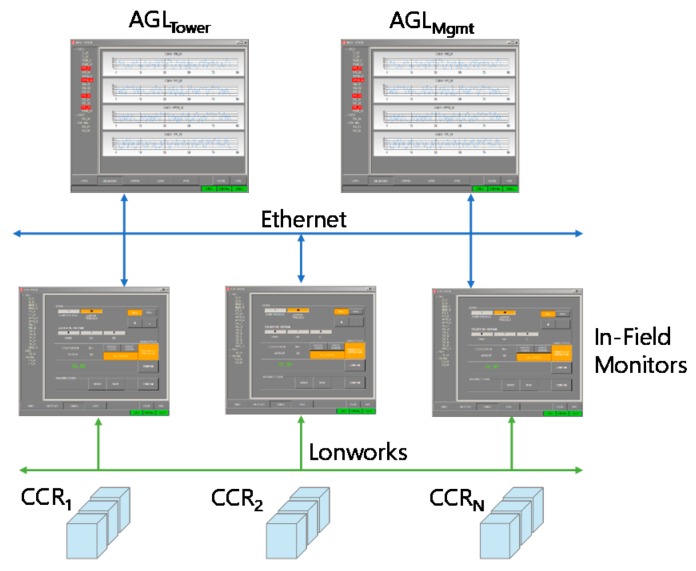
The IRMS system.

**Figure 4:**
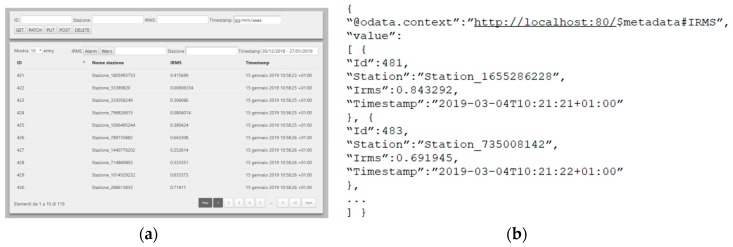
Examples of Web Logger GUI (**a**) and JSON response (**b**) for the Insulation Resistance Monitoring System (IRMS).

First of all, we have upgraded the legacy Insulation Resistance Monitoring System (IRMS) in charge of identifying any light fault in the airfield by comparing input and output tensions within a closed circuit (see Figure 3). The field bus is still based on the LonWorks protocol to dispatch events from CCR units to in-field monitors (Figure 3, down). On the contrary, the ethernet part pushing information to the AGL for both the tower control and the maintenance stations (Figure 3, up) is now based on AMQP instead of the traditional socket-based proprietary protocol. Also note that we have been able to integrate the new messaging system (and thus dismiss the previous socket-based communication protocol) while not modifying the rest of the application. In particular, we did not need to modify the previous GUI, thus providing in-field monitor and AGL users with a consistent user experience. In addition, we adopted the Web Logger to present IRMS monitored information to users via a novel HTML-based Web GUI (Figure 4a) and to dispatch information as JSON documents based on the OData standard (Figure 4b).

To verify responsiveness, reliability, and scalability, we have considered the challenging scenario of stop bars. In this scenario, the reliable and prompt delivery of commands (e.g., to turn on/off stop bars) as well as sensed data (e.g., to rise an alarm in case an airplane does not stop at an active stop bar) is mission critical. Current international regulations enforce the dispatching of failure events in, at most, 2 s. However, 1.5 s are typically required for the in-field part, thus the actual dispatching of states and commands based on IoT standards from in-field monitors to the AGL can last 0.5 s at most. 

We have tested the responsiveness of our prototype while dispatching an increasing amount of messages per second by measuring the end-to-end delay from alarm dispatching by in-field monitors to its visualization on a Web browser. To stress its scalability, tests were performed increasing the number of messages from 1 to 1000 per second, with a message payload of 1 KB, and limiting the available bandwidth to 10 Mbit/s. Each test lasted 5 min, while nodes were equipped with a 2.6 GHz quad-core, 8 GB RAM, Linux Ubuntu 18.04 or Windows 10 operating system.

**Figure 5:**
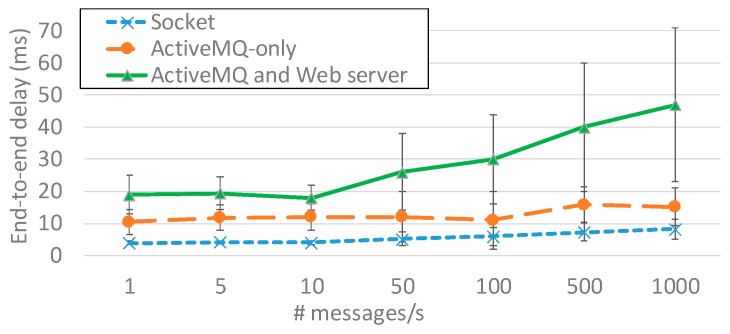
End-to-end delay average and standard deviation with socket, ActiveMQ-only, and ActiveMQ and Web server.

Figure 5 shows results comparing three different solutions: (i) the traditional socket-based solution, (ii) the ActiveMQ-only one (broker version 5.15.x, client library based on ActiveMQ NMS 1.7.2 version) exploiting the StompIt JavaScript Node.js library (0.26.0 version) within the Web browser to receive STOMP messages, and (iii) the solution also considering the Web server (based on NET Core 2.1 SDK and NET Framework 4.6 SDK) in charge of receiving AMQP messages and sending them via WebSocket. As Figure 5 shows, the socket-based solution presents the best end-to-end delay, with just 8 ms in the case of 1000 messages per second. The ActiveMQ-only solution provides slightly worse end-to-end delay, with a maximum of about 15 ms in case of 1000 messages per second. Finally, the solution comprising both ActiveMQ and the Web server further increased the end-to-end delay by up to 43 ms (with higher standard deviation) in the case of 1000 messages per second. 

Let us note that notwithstanding the observed increasing trend, achieved delay is limited in every tested case, thus demonstrating the suitability of the proposed solution in relation to temporal constraints of airfield environments. In other words, the adoption of standard and ease of adopting communication protocols allowed us to greatly increase extendibility and flexibility (as described in previous sections), while imposing a limited overhead that was well below imposed constraints. In addition, note that achieved delays represented an upper bound, since the expected bandwidth is greater than 10 Mbit/s and in the target environment the expected message frequency is usually much lower, varying in relation to system complexity but in the order of at most few hundred messages per second. 

Focusing on reliability and fault-tolerance, we tested an ActiveMQ broker cluster [28] with 2 replicas. For this purpose, publishers and subscribers connect to replicas in a round-robin fashion by exploiting the failover mechanism with randomize = false option, i.e., IP addresses of replicas are statically provided to nodes and they always connect to the first replica of the list, to other replicas only in case the first one fails. Note that in these tests we aimed at verifying the behavior of proposed failover mechanisms in a testing environment similar to the deployment one rather than its scalability. For this reason, we adopted a smaller payload (only few bytes rather than 1 KB) and no bandwidth limitation (rather than a 10 Mbit/s limit), thus achieving smaller end-to-end delays. 

**Figure 6:**
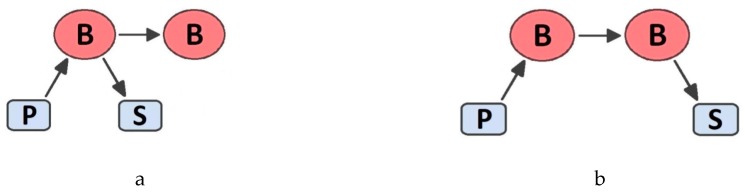
Tested cluster architectures: clients connected to the same (**a**) and different (**b**) replicas.

We tested two different cluster architectures, one in case both the publisher and the subscriber are connected to the same broker replica (Figure 6a) and the other in case the publisher and the subscriber are connected to different broker replicas (Figure 6b). In addition, we have tested with and without the ActiveMQ durable subscription feature [29]: if activated, the broker persists in sending messages for a while, to deliver them to subscribers in case they connect after a message has been dispatched.

Table 1 shows the number of lost messages when abruptly stopping the broker replica the subscriber is connected to while sending an increasing amount of messages per second. It is worth noting that without durable subscriptions and with only with 100 messages per second, there are few lost messages, while higher message frequencies increase the number of lost messages. 

In case clients are connected to the same fault replica, both the subscriber and the publisher switch to the other replica and the latter stops sending messages while switching. However, some messages are lost, since the subscriber does not receive messages the publisher has recently delivered to the broker. Also note that in case of the publisher and the subscriber being connected to different broker replicas, the outcome is slightly worse. In this case the publisher does not perceive the replica failure and keeps sending messages (not delivered to the subscriber) while the subscriber is also reconnecting to the other broker replica. Let us stress that achieved results do not depend on the amount of adopted replicas, since replicas communicate one each other via multicast and thus additional replicas do not increase the generated traffic. Instead, as noted above, performance results vary if the publisher and the subscriber are connected either to the same or different replicas. Finally, it is possible to avoid any message loss (even at very high message frequencies) by adopting durable subscriptions, at the cost of slightly increasing the end-to-end delay (about 1 ms in our tests).

**Figure 7:**
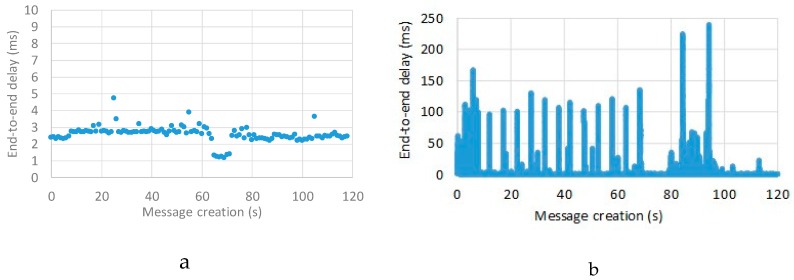
End-to-end message delivery delay of two 120 s long experiments with message creation frequency set at 1 (**a**) and 750 (**b**) messages per second: broker cluster configuration with two replicas, a replica fails at 60 s and another starts at 70 s.

To better understand the behavior of our solution at replica failure, Figure 7 presents the end-to-end delay of packets during two different experiments. Both experiments lasted 120 s and in both cases a replica failed after 60 s and then a new replica started after 10 s. Replicas were configured with durable subscriptions, with the publisher and subscriber connected to the same replica. In the first experiment (Figure 7a) message creation frequency was set to 1 message per second, in the second experiment (Figure 7b) message creation frequency was set to 750 messages per second.

It is worth noting that at very low message frequency (1 message per second, Figure 7b) when a replica fails (at about 60 s) the end-to-end delay lowers, since the overhead due to message dispatching towards different replicas is temporarily removed. Then, the previous end-to-end delay is achieved when a new replica joins the cluster (at about 70 s). With much higher message frequency (750 messages per second, Figure 7b) the time delay variability increases, i.e., message delay more relevantly varies from message to message. In addition, delays are higher for a while right after the subscriber has switched to the active replica, since at 750 messages per second there are many messages saved in the still active broker replica (thanks to the durable subscription feature) and waiting to be dispatched to the subscriber. Then, once saved messages are delivered, the average delay stabilizes at lower values, since both the publisher and the subscriber are now connected to the same broker replica, thus avoiding inter-replica delays.

In conclusion, presented performance results demonstrate the proposed solution not only greatly increases the interoperability of the legacy lighting system by adopting IoT standards, but it also provides fault tolerance without relevantly reducing the performance of end-to-end delay dispatching, even in the case of exploitation of both ActiveMQ and Web server. It is also worth noting that the adoption of IoT related technologies allows us to more easily add new replicas, e.g., by running a new ActiveMQ broker instance and adding it to the cluster even during service provisioning. In addition, it allows us to more easily adopt secure connections, e.g., enabling TLS/SSL on brokers and Web servers. However, technicians should careful to identify the best tradeoff among replication degree, security, and required performance, by considering that higher levels of replication/security may negatively impact on end-to-end latency.

## 5. Conclusions

The ever-increasing demand for easier integration with third-party software solutions has imposed a new approach for developing airport ALCMS solutions. This paper demonstrates how the adoption of an open and IoT approach based on well-known standards can pave the way for more open monitoring and control solutions that support not only easy integration but also non-functional requirements such as availability, scalability, and security. The developed technical solution allows the integration of third-party software and hardware components by developing AMQP publishers/subscribers, interacting via STOMP, or invoking REST-based OData API. Moreover, performance results demonstrate that a two-replica message broker cluster with durable subscriptions allows the guarantee of no messages being lost even while publishers and subscribers switch to another broker replica, while limiting the end-to-end delay.

The encouraging results already achieved, based on a working solution, are stimulating our ongoing research work. We are mainly working on the development of security features to enforce fine-grained authentication and authorization access to topics and information, also to guarantee accountability of issued commands. We are also developing an Android application to provide up-to-date monitoring information to technicians in a ubiquitous manner.

## Figures and Tables

**Table 1:** Lost packets at broker failure.

Durable	Connected Replica	Messages Per Second						
		1	10	20	100	250	500	750
no	the same	0	0	0	1	8	15	30
	different	0	0	0	1	8	23	70
yes	the same	0	0	0	0	0	0	0
	different	0	0	0	0	0	0	0

## Abbreviations

The following abbreviations are used in this manuscript:

IoT	Internet of Things
REST	REpresentational State Transfer
AMQP	Advanced Message Queuing Protocol
IT	Information Technology
AGL	Airport Ground Lighting
ALCMS	Airport Lights Control and Monitoring System
RVR	Runway Visual Range
FAA	Federal Aviation Administration
CCR	Constant Current Regulator
PAPI	Precise Approach Path Indicators
SNS	Sensor
STB	Stop Bar
AUX	Auxiliary
UPS	Uninterruptible Power Supply
FLS	Flash
GUI	Graphical User Interface
AFS	Application Field Server
API	Application Programming Interfaces
IRMS	Insulation Resistance Monitoring System
